# Valve-in-Valve Transcatheter Mitral Valve Replacement in a Very High-Risk Octagenerian Patient: A Case Report

**DOI:** 10.7759/cureus.61493

**Published:** 2024-06-01

**Authors:** Charalampos Kakderis, Matthaios Didagelos, Antonios Kouparanis, Vasileios Kamperidis, Antonios Ziakas

**Affiliations:** 1 Department of Cardiology, AHEPA Hospital, Aristotle University of Thessaloniki, Thessaloniki, GRC

**Keywords:** structural heart diseases, octagenerian patient, degenerated bioprosthetic valve, transcatheter mv replacement, valve-in-valve

## Abstract

Degeneration of the surgical bioprosthetic valves remains one of the most common complications of surgical valve replacement. Redo surgery is the gold standard, but unfortunately, most of these patients are deemed inoperable because of the high perioperative mortality. Transcatheter implantation of a new valve inside the degenerated bioprosthesis (valve-in-valve (ViV)) has emerged as an alternative solution. A 79-year-old patient with a medical history of surgical replacement of the mitral valve with a bioprosthetic valve, coronary artery bypass graft surgery (CABG) with implantation of the left internal mammary artery (LIMA) to the left anterior descending artery (LAD), paroxysmal atrial fibrillation, and chronic kidney disease was referred to our hospital for ViV transcatheter mitral valve replacement (TMVR). He had recent hospitalizations with pulmonary edema caused by severe stenosis of the bioprosthetic valve and his perioperative mortality for a redo surgery was very high (EuroSCORE II: 13.72%). The ViV TMVR was performed with a transseptal approach and after the implantation of the new valve, the mean pressure gradient was dropped from 19.39 to 2.33 mmHg. The procedure was technically successful and the patient was discharged asymptomatic.

## Introduction

The progress of percutaneous treatments for structural heart disease is remarkable, especially regarding the aortic valve. Transcatheter heart valve replacement can be performed in both native valves and in failed bioprostheses. The balloon-expandable aortic transcatheter heart valves can therefore be implanted inside the degenerated bioprosthetic mitral valves (valve-in-valve (ViV)), especially in patients who are deemed inoperable for a second surgery. It is estimated that 34% of the surgical bioprosthetic mitral valves will be degenerated in the first 10 years and a new operation will be required [1]. Transcatheter mitral valve replacement (TMVR) can also be performed in patients with mitral annular calcification (MAC) and in failed mitral annuloplasty rings [2], but the best results in all-cause mortality are highlighted in the ViV TMVR [3]. The first operations were performed transapical but the transseptal approach soon prevailed. The results of the transseptal ViV-TMVR are very encouraging and in recent studies, one year after the procedure, 89.3% of patients remained in New York Heart Association (NYHA) functional class I or II [4]. We present a case of ViV TMVR in an inoperable patient who was successfully treated with a percutaneous intervention.

## Case presentation

**Figure 1:**
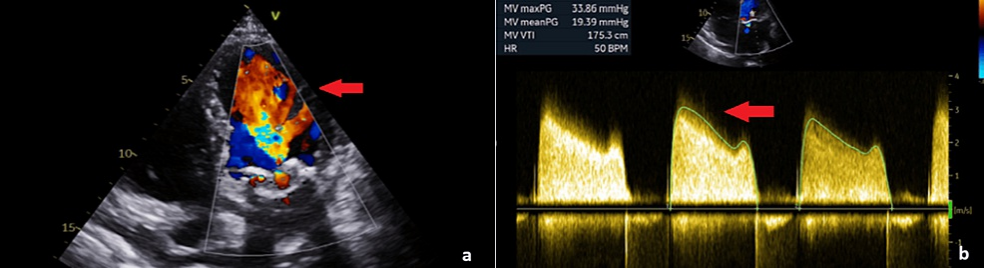
a. Color Doppler in the echocardiogram with accelerated velocities through the bioprosthetic valve (red arrow); b. Severe stenosis of the bioprosthetic valve with a meanPG 19.39 mmHg (red arrow).

A 79-year-old male patient was admitted to our unit for a planned ViV transcatheter mitral valve replacement (ViV TMVR). He had a medical history of paroxysmal atrial fibrillation, a previous coronary artery bypass graft surgery (CABG) with implantation of the left internal mammary artery (LIMA) to the left anterior descending artery (LAD), and chronic kidney disease. He also had a surgical replacement of the mitral valve (MV) with a 31 mm Carpentier-Edwards Perimount Magna bioprosthesis (Edwards Lifesciences, Irvine, United States) nine years ago. The patient was in NYHA III class and the bioprosthetic valve degenerated showing severe stenosis with a mean pressure gradient of 19.39 mmHg and a borderline left ventricular injection fraction (LVEF) of 50% with severe pulmonary hypertension (PASP > 55 mmHg) (Figures 1a, 1b).

**Figure 2:**
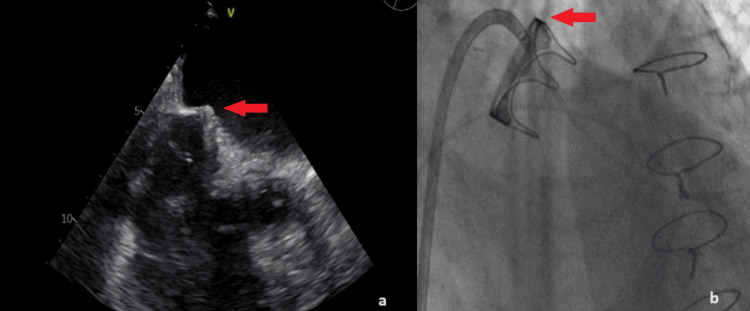
a. BRK1 Transeptal needle (St. Jude Medical, Saint Paul, United States) piercing the interatrial septum (red arrow); b. The Agilis sheath (Abbott, Chicago, United States) steered towards the left ventricular apex (red arrow).

Because of the high perioperative mortality of a redo surgery (EuroSCORE II: 13.72%) the patient was referred for ViV TMVR. Before the procedure, his regular anticoagulation scheme with low molecular weight heparin (enoxaparin) was suspended for several days because of a spontaneous hematoma in the iliopsoas muscle of his left leg. He received several blood transfusions and was successfully stabilized. A transesophageal echocardiogram (TOE) was also performed and excluded the existence of a thrombus in the left atrial appendage. The procedure was performed under general anesthesia with TOE guidance. Bilateral common femoral veins and the right femoral artery were accessed with 6 French (Fr) sheaths. A pigtail catheter was advanced in the non-coronary cusp of the aortic valve to serve as an anatomic landmark. A temporary pacemaker was then inserted through the left vein femoral sheath and anchored in the apex of the right ventricle. Under fluoroscopy, the transeptal sheath with the dilator entered the right atrium (RA). A BRK1 transeptal needle (St. Jude Medical, Saint Paul, United States) was advanced through the transeptal sheath and directed toward the fossa ovalis. Under 3D-TOE and fluoroscopic guidance, the atrial septum was punctured (Figure 2a). The sheath was advanced in the left atrium (LA), the transeptal needle was withdrawn and an Inoue wire was introduced in the LA. Over the Inoue wire, an Agilis NxT Steerable Introducer sheath (Abbott, Chicago, United States) was placed in the LA and steered toward the left ventricular apex (Figure 2b).

**Figure 3:**
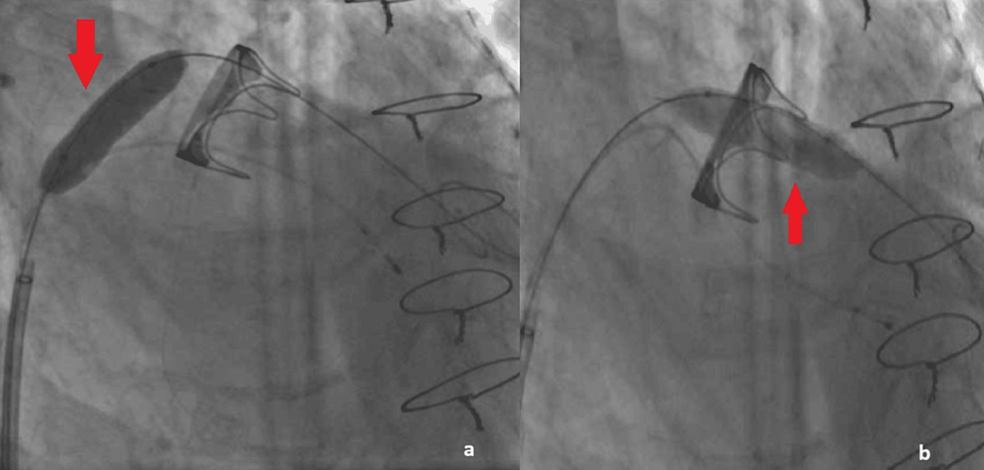
a. The interatrial septum was dilated with an OTW 12x40 mm Oceanus 35 balloon (red arrow), b. The degenerated bioprosthetic valve was also predilated with the same balloon (red arrow).

A Swan-Ganz catheter was inserted in the LV through the Agilis sheath with the balloon inflated to prevent any chordal rupture and a J-wire was put in the apex through the pulmonary catheter. A pigtail catheter was then advanced over the J-wire and through the pigtail catheter a Confida Brecker Guidewire (Medtronic, Minneapolis, United States) was safely positioned in the LV. Over the Confida wire (Medtronic, Minneapolis, United States) a 14 Fr expandable introducer sheath Python (Meril Life Sciences Pvt. Ltd., Gujarat, India) was inserted. The interatrial septum was then dilated with an over-the-wire (OTW) 12x40 mm Oceanus 35 balloon (iVascular, Barcelona, Spain) and the degenerated bioprosthetic valve was also predilated (Figures 3a, 3b).

**Figure 4:**
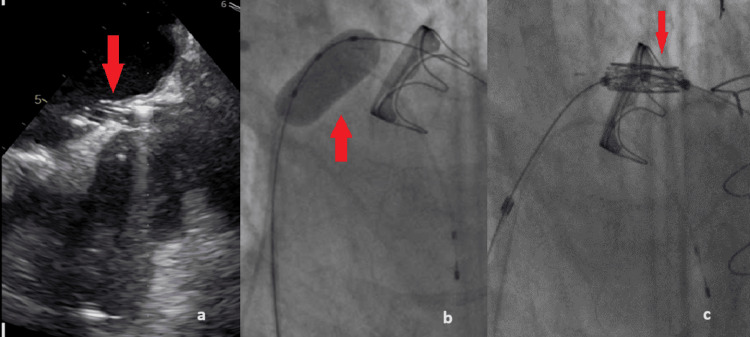
a. Even though the interatrial septum was dilated the transcatheter balloon-expandable heart valve Myval (red arrow) could not pass through; b. A new balloon, the 16x40 mm Mammoth (red arrow) was used to perform a second dilatation of the septum; c. Myval (red arrow) is positioned inside the degenerated valve.

The balloon was then withdrawn but unfortunately, the transcatheter balloon-expandable heart valve Myval (Meril Life Sciences Pvt. Ltd., Gujarat, India) could not cross through the interatrial septum (Figure 4a). A new balloon, the 16x40 mm Mammoth (Meril Life Sciences Pvt. Ltd., Gujarat, India) was used to perform a second dilatation of the septum (Figure 4b), and then the Myval (Meril Life Sciences Pvt. Ltd., Gujarat, India) successfully crossed and positioned inside the degenerated bioprosthesis (Figure 4c).

**Figure 5:**
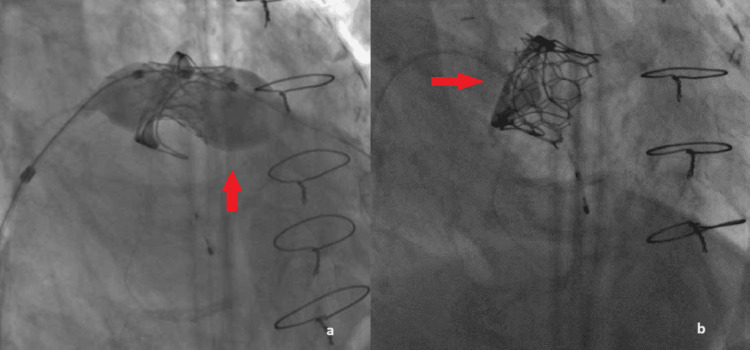
a. The new valve was deployed inside the degenerated bioprosthesis under rapid ventricular pacing (red arrow); b. The new valve after successful implantation (red arrow).

Under rapid ventricular pacing the new valve was deployed (Figures 5a, 5b).

**Figure 6:**
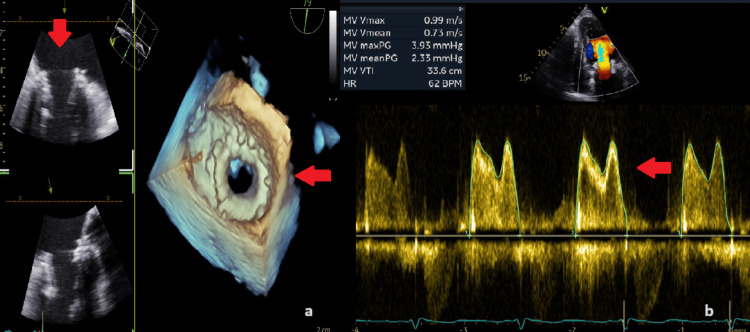
a. TOE confirmed successful implantation of Myval (red arrows); b. The mean pressure gradient after the implantation was 2.33 mmHg (red arrow).

TOE was immediately performed and confirmed the successful implantation, the good motion of the leaflets with no paravalvular leak (Figure 6a), and a mean pressure gradient of 2.33 mmHg (Figure 6b). The patient was then transferred to the coronary care unit (CCU) and was extubated. Unfortunately, he had a long hospitalization because of pneumonia, acute severe kidney disease, and iliopsoas hematoma relapse, but was finally successfully discharged.

## Discussion

ViV TMVR represents an effective and safe solution for the treatment of failed bioprosthetic valves in patients who are deemed at very high risk for surgical re-intervention. Although the procedure is not as prevalent as the percutaneous replacement of the aortic valve, it remains a viable alternative for the treatment of the degenerated mitral bioprothesis. The first procedures were performed transapical (TA) with a small thoracotomy, but the transseptal (TS) approach prevailed. Patients with the TA procedures had longer hospitalization in the intensive care units and more overall hospitalization time than the patients treated with the TS mitral valve replacement [5]. With the progression of the introducer sheaths and the catheters that are used, procedural technical success has risen to 94-98% [6]. Recent studies have also highlighted the safety of the procedure with all-cause mortality rates at one month and at one year ranging from 93-97% and 83-89%, respectively [6]. Although the transcatheter replacement has been established as a safe procedure, complications can occur. The most common are left ventricular outflow tract obstruction (0-6%), valve migration (0-9%), and residual regurgitation (0-6%) [7]. TMVR can also be performed in patients with severe mitral annular calcification (valve in MAC) and failed mitral annuloplasty ring repair (mitral valve in ring). The results though, are inferior to the ViV replacement with higher two-year-all cause-mortality rates of both procedures [3]. In comparison to the redo mitral surgery, the percutaneous mitral ViV replacement has proven to be equally effective. Mortality rates with the redo operation against TMVR are statistically similar at one year (11.3% versus 11.9%, p = 0.92), and the percutaneous intervention results in fewer bleeding complications and arrythmias [7].

## Conclusions

ViV TMVR is a safe and effective solution for inoperable patients with degenerated bioprosthetic mitral valves. In this case, we would like to highlight this procedure as the optimal option for these patients. Percutaneous treatment in high-surgical-risk patients represents the future in the management of structural heart disease.
